# Evaluating Dynamic Approaches to Key (Re-)Establishment in Wireless Sensor Networks

**DOI:** 10.3390/s19040914

**Published:** 2019-02-21

**Authors:** Lukas Nemec, Vashek Matyas, Radim Ostadal, Petr Svenda, Pierre-Louis Palant

**Affiliations:** 1 Centre for Research on Cryptography and Security, Faculty of Informatics, Masaryk University, Brno 60200, Czech Republic; lukas.nemec@mail.muni.cz (L.N.); ostadal@mail.muni.cz (R.O.); svenda@fi.muni.cz (P.S.); 2 Institut National des Sciences Appliquées Centre Val de Loire, 18000 Bourges, France; pierre-louis.palant@insa-cvl.fr

**Keywords:** adaptive, autonomous, cryptography, distributed, protocol, security, wireless radio communication

## Abstract

Wireless sensor networks with a large number of cheap low-power interconnected devices bring up challenging tasks when considering the security of their communications. In our previous work, we presented two approaches for the design of dynamic protocols for link key (re-)establishment in ad hoc networks, using two elements studied earlier—secrecy amplification and key extraction from radio channel fading. The goal of this article is to provide a unified approach to the design of these protocols, together with their experimental verification, in a real network with various settings. The overall results of our experiments show that our dynamic combination of secrecy amplification and key extraction from radio channel fading saves a significant portion of messages with corresponding energy expenditure and can adapt to a much wider scale of environments when compared to previous solutions based on the exploitation of the individual elements of secrecy amplification and key extraction from radio channel fading.

## 1. Introduction

With the rise of IoT (Internet of Things) and growing numbers of interconnected devices, the research in the area of wireless sensor networks (WSNs) is becoming significantly more relevant. The research done in the early 2000s focused on small, low power devices can now be directly applied to most IoT networks. Overall, the WSNs and IoT have many common features, including limitations in terms of energy, computation power, and storage capacity. In addition, in both, we assume no tamper resistance against physical attacks, as the price of individual devices does not permit it. In addition, both types of systems sense, process and share sensitive data, which introduces the requirement of secure communication between individual devices.

In our research, we focus on key distribution in general ad hoc networks, as the key distribution can be considered a fundamental building block to secure communication. We assume use of (communication) link keys for every connection in between individual devices. However, we make no assumptions about the link keys themselves. In fact, we assume that the attacker has compromised the network to a certain extent and has the knowledge of a certain fraction of these link keys.

The main goal of our protocols is to re-secure a partially compromised network, without any assumption on the network environment. We are using the secrecy amplification (SA) protocols developed in the WSN era, combined with the key extraction (KEx) from radio channel fading. The combination of two can achieve better overall performance and possibly solve the problematic edge cases, which otherwise would be difficult to work with.

An SA protocol describes the way a group of neighboring nodes in a partially compromised network cooperates together to re-secure compromised link keys. To secure a given communication link in between two neighbors, they select several common neighbors, which they use to deliver key updates through, without using the compromised link. Previous research [1] showed the ability of SA protocols to improve the security of the network from 50% of compromised link keys to 90% of secure links after SA protocol execution. The exact results depend on a particular attacker model, the initial key establishment protocol, and the particular SA protocol being used.

The KEx utilizes the radio channel properties to generate secret bits shared between two radio-enabled devices. The amount of secret bits is highly dependent on the environment (e.g., the movement of an object in the area between the two neighbors creates disturbances) and also on the number of messages exchanged between these two nodes. KEx does not require any secured channel, only public messages are being exchanged. This can be achieved thanks to properties of radio channel fading, which decorrelates over $\frac{\lambda }{2}$ (half the wavelength of the signal at the used broadcasting frequency). Combining the fading phenomenon and disturbances in the radio channel, the generated bits are unique to the time and position of these particular nodes.

In our previous work [2], we have combined SA and KEx into a particular dynamic protocol that can adapt to the network environment. Shifting the ratio in between the SA and KEx usage can make the protocols fit into various network environments. As we do not know what links in the network were compromised by the attacker, we have no way to estimate the success rate of SA execution. However, using the min-entropy estimates [3], we can measure the outcome of KEx on any given link. The SA is therefore used only for links with a low amount of entropy gathered by KEx.

The other approach we researched in [4] assumes that the network environment is not suitable for KEx execution. However, we aim to utilize even the small amounts of entropy produced. The two protocol versions (Push and Pull) are inspired by the design of a simple secrecy amplification protocols [5]. Using a common neighbor of two nodes, we combine the entropy gathered over multiple connections in the network, producing one secure link. As shown in the results of individual SA protocols performance [1], every single secured link can make a considerable impact on the result itself.

The main goal of this article is to provide a holistic view of the novel protocol design. The key contributions are:

- extension of the protocol design for dynamic key (re-)establishment in WSNs;
- verification of the design in a real testbed and in several environmental and network conditions.

The roadmap of our article is as follows. Section 2 reviews the concept of secrecy amplification, its basic protocols, as well as key extraction from radio channel fading and key management schemes for WSNs. Our attacker model is presented in Section 3. Dynamic approach protocols in their basic versions are described in Section 4. The individual protocol parameters and testbed setup are described in detail in Section 5. The results from our experiments are described in Section 6, the impact, and conclusions from our experiments are discussed in Section 7.

## 2. Previously Used Methods

### 2.1. Secrecy Amplification

The SA concept was originally introduced in [6] to improve the security of the WSN where the link keys were initially established using the plaintext key exchange among neigbourings nodes, but the technique can be used to improve the overall security of any partially compromised network. Let the link key shared between nodes *A* and *B* be compromised by the attacker. If a secure path between nodes *A* and *B* exist (no link key is compromised on that path by the attacker), we are able to deliver a key update using the secure path and we can re-secure the originally compromise link key.

The trivial approach would be to utilise every possible path between every pair of neighbouring nodes in the network. However, this is not feasible due to the huge communication overhead (communication is energy intensive operation) for nodes with limited resources and limited energy. The SA protocol defines the way how the group of neigbouring nodes cooperates and the way how different paths are selected for key updates delivery.

**Figure 1:**
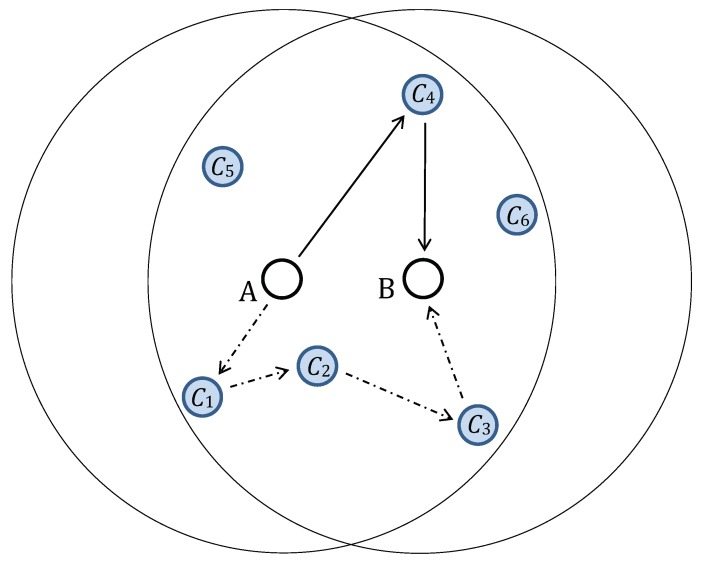
Two paths selected for a key update transmission from node *A* to node *B*. The solid lines mark a delivery path with a single selected intermediate node, $C_{4}$, and the dash-dotted lines mark a delivery path with three intermediate nodes, $C_{1}$, $C_{2}$, and $C_{3}$.

The overall aim is to utilise SA protocols that can re-secure a high number of links yet require only a small number of messages and are easy to execute and synchronise in parallel executions in the large ad hoc network. Figure 1 provides an example of two simple SA protocols. The principal steps of SA protocol follow:

1. Selection of neighbouring nodes participating in a given SA protocol. The link key between nodes *A* and *B* is being updated and $C_{i}$ denotes intermediate nodes used to form the key updates delivery paths.
2. Generation of fresh key updates. Node *A* generates key updates in both examples in Figure 1.
3. Determination of delivery path and transportation of generated key update.
4. Combination of key update and existing old key into a new link key with an appropriate one-way function. Then, the new key will be secure if either the old key is secure or the key update was delivered through a secure path.

Three distinct classes of SA protocols were identified during the research, each with different properties, advantages, and disadvantages. We provide a brief description of those classes:

- *Node-oriented protocols* send key updates via every possible neighbour or neighbours by a simple protocol. The node-oriented protocol is executed for all possible *k*-tuples of neighbours in the network. The protocol is very simple and easy to synchronise in large networks. The biggest disadvantage of node-oriented protocols is a high communication overhead. The number of messages increases polynomially with respect to the number of neighbouring nodes and exponentially with respect to the number of parties participating in the protocol (the number of intermediate nodes along every path from node *A* to node *B*).
- *Group-oriented protocols* were proposed to improve the communication overhead of node-oriented protocols. The group-oriented protocols share key updates inside a bigger group of cooperating nodes identified by their geographical areas in the form of relative distance to selected nodes. Even the protocols succeeded to reduce the communication overhead; the protocols pose several disadvantages—the challenge of synchronising parallel executions and the complexity of the security analysis due to the high number of nodes involved. Group-oriented protocols were never implemented apart from simulation environment due to the described complexity.
- *Hybrid designed protocols* use sub-protocols (similarly to node-oriented), relative distances (similarly to group-oriented) and additionally utilize several repetitions of the whole process to achieve required success rate. Hybrid designed protocols combine advantages from both previous approaches and eliminate most of the disadvantages. Hybrid designed protocols are very simple, synchronization of parallel executions is possible, require a low number of messages and are easy to analyse and implement.

Secrecy amplification protocols were shown to be very effective, yet for the price of a non-negligible communication overhead. Any solution with lower communication overhead, yet with the same efficiency in the form of a number of re-secured links is desirable.

### 2.2. Key Extraction

Key extraction from radio channel fading uses the natural phenomena of radio wave propagation to generate randomness. The process itself is rather simple and straightforward. The theoretical background was provided by Maurer and Wolf in [7], which was several years later independently picked up by few different researchers [8,9,10], each one of them providing their own algorithm suitable for a slightly different network environment.

The common building blocks of these algorithms are a collection of radio channel fading data (commonly used is RSS—received signal strength) and quantization (transformation of RSS measurements into bits). For the first part, we usually prefer to collect RSS measurements from a regular network traffic, as creating artificial messages just to collect RSS data would not be wise due to the limited resources of common WSN/IoT devices. The process itself then requires either full duplex radio modules (which are not commonly available) or use of any off-the-shelf half-duplex radio module and near to instant acknowledgments to the messages. Two communicating parties then can exchange series of messages, one side recording the RSS of the original messages, the other party recording the RSS of acknowledgments for these messages. The series of measurements obtained on both sides cannot be exactly the same; however, thanks to the almost instant acknowledgments, the produced sequences are highly correlated. The series of such RSS measurements are the main input for all of the algorithms that ensure matching output for both parties.

Quantization of the RSS data comes in two distinct approaches, lossless and lossy. The name refers to the amount of measurements corresponding to the amount of bits on the output—whether every RSS measurement is processed down to a single bit in the output or whether some RSS measurements can be dropped, making the resulting bit sequence shorter.

From evaluation experiments provided by Suman Jana et al. [9], we can estimate suitability of each one of these different algorithms for different environments. All algorithms expect the same input—a sequence of RSS measurements; however, the processing into bits (quantization) offers many different routes to choose. The quantization process which we use in our protocols, proposed by Suhas Mathur et al. in [8] and improved by Suman Jana et al. in [9], works as follows:

- Calculate the mean *m* and standard deviation sd of the sequence of RSS measurements.
- Calculate the thresholds $q^{+}=m+sd*\alpha$ and $q^{-}=m-sd*\alpha$, where the α can range from 0 to 1, depending on the network environments and the required setup.
- Process all RSS measurements using the thresholds $q^{+}$ and $q^{-}$, where measurements above the $q^{+}$ result in bit value 1 appended to the output sequence, measurements below the $q^{-}$ result in value 0. The values in between are dropped.
- Both participating parties publicly exchange the set of indices for non-dropped measurements. For the final sequence, only indices within the intersection of such sets are used.
- Both parties use the resulting bitstring to (re-)secure communication channel in between them.

In favorable environments, any of the algorithms can provide a sufficient amount of entropy in a reasonable time. However, none of them will work for all the possible environment settings. The major issue for any algorithms is a static network with a low amount of disturbances. Such an environment is not suitable for deployment of these algorithms at all. The main culprit is low to no entropy output, which is to be expected given the nature of such environment. Nevertheless, one would like to secure any environment, especially the problematic ones.

### 2.3. Key Management Schemes—State of the Art

Key management schemes (KMSs) deal with a rather difficult task of establishing secure communication channels in between individual nodes (sometimes also called motes) in the wireless sensor network. Such secure channels allow for message exchange in a safe manner, even in the presence of an attacker. As such a task is very complex, many solutions have been proposed; in this section, we cover those with major impact on the development of KMSs.

Extensive surveys of existing approaches to KMSs are presented in [11,12,13,14].

The network-wide master key pre-distribution is usually the most simple scheme considered. Every node in the network receives the same master key prior to the deployment, and this master key is used to secure communication across all links in the network. This particular scheme excels with the efficiency, mostly regarding the memory used. From the security standpoint, it is not the optimal solution, as this scheme fails completely when the master key is compromised.

On the completely opposite side of the KMS spectrum is the pairwise key pre-distribution. Every node is equipped with a unique key to every other node in the network. From the security perspective, it offers almost optimal security properties, as a single key compromise affects only one link. However, in WSNs and similar networks, it is not feasible to store n−1 keys on every node (assuming a network of *n* nodes), due to many constraints that these devices have.

#### 2.3.1. Master Key Based Derivatives

Localized encryption and authentication protocol (LEAP) [15] is a scheme that builds upon the master key pre-distribution principle, which uses several types of keys for each node in the network. Once a pre-distributed master key is shared in between all members of the network (the group key), *n* pre-distributed individual keys are shared in between the base station and individual nodes. In addition, pairwise keys in between neighboring nodes are derived from the pre-distributed master key after neighbor discovery phase. Finally, the master key is erased from the node memory once the protocol is completed. Authors argue that the time required to establish such pairwise keys after deployment is not sufficient for the attacker to successfully retrieve such a master key from the node internal memory.

A similar approach to create pairwise shared keys is used by authors of BROSK [16], who use broadcasted key negotiation messages and message authentication code (MAC) to compute shared keys. Contrary to LEAP, the master key is not erased after the key establishment phase.

#### 2.3.2. Trusted Third Party Based Derivatives

Several approaches assume the presence of a central authority (base station) that assumes a position of the trusted third party and negotiates the key establishment in between two nodes. Example of such an approach is the SPINS [17] protocol. However, the central point (base station in case of the SPINS protocol) becomes the single point of failure. A possible solution to this problem is offered by the authors of the PIKE [18] protocol, where every pair of neighboring nodes have their own trusted third party, thus removing the need for a central entity in the network.

#### 2.3.3. Pairwise Key Based Derivatives

A clever twist on the pairwise key distribution comes with the probabilistic key pre-distribution presented in [19]. Prior to the deployment, a pool of *P* keys is generated, and every node receives a ring of *R* keys with identifiers (*P* should be significantly greater compared to *R*.), randomly selected from the pool. After the deployment, neighbors can openly negotiate what keys they share with each other and establish a shared set of keys, if any exists. *P* and *R* can be adjusted, so that the probability that two nodes share the required amount of keys has the required value. Several concepts extend this scheme [20,21].

#### 2.3.4. Eliptic Curve Cryptography Derivatives

Three key exchange protocols derived from the Diffie–Hellman key exchange—that has been used as a basis for cryptographic protocols with different purposes as confidentiality, authentication, or key exchange—are proposed in [22]. The authors used elliptic curves version of the Diffie–Hellman method, which allows for a significant decrease of key length and computing capabilities—making the algorithm suitable for devices with limited capabilities.

Another approach to the problem of limited resources is the use of a dedicated cryptographic co-processor, as did the authors of [23].

## 3. Attacker Model

In our protocol design, we assume a passive attacker—a composition of commonly used attacker models for both SA [19,24] and KEx [9]. We also define two phases of attacker activity—initial network compromise and attacker behavior during the standard network operation, after the initial compromise. We also assume that the attacker knows all algorithms and parameters used for both SA and KEx.

### 3.1. Initial Network Compromise

As per the initial network compromise introduced in [19], we expect that every pair of neighbors shares a unique link key in the node compromise attacker model. The attacker is able to capture a fraction of deployed nodes as no physical control over deployed nodes is assumed. The attacker is able to extract keying material from every captured node. After the extraction, the node operates in a standard way and the attacker has no control over the node.

### 3.2. Attacker Capabilities during the Network Operation

We assume a global passive attacker that can eavesdrop on all communication within the area of reception, considering the transmission power used by regular nodes.

With respect to SA, the goal of the attacker is to compromise an updated key $K^{\prime }$, where the original key is *K* (has to be already compromised) and the nonce *N* was used for the key update. To do so, the attacker has to eavesdrop at least one SA message with the nonce *N* and the attacker has to be able to decrypt this message—the link key used to protect the message has to be already compromised.

With respect to KEx, the attacker can measure the channel between herself and the node of origin for every eavesdropped message, regardless of used link key (compromised or non-compromised). The attacker is able to determine the secret bit generated during KEx, assuming the distance between the attacker and legitimate communicating nodes is shorter than half the wavelength, which we allow, contrary to the usual KEx attacker model.

## 4. Dynamic Approach Protocols

### 4.1. Entropy Driven Secrecy Amplification

Our proposal in [2] presents a combination of both SA and KEx, which is designed to work in any environment. The protocol itself leverages already existing network traffic to collect RSS measurements for all links in the network. Upon a trigger (based on time or amount of messages), the KEx quantization process is executed for a given link in the network, resulting in a bitstring. Consequently, we execute the min-entropy estimate test suite [3] to obtain a conservative estimate of the entropy within the computed bitstring.

Based on the entropy estimate, we either secure the link directly, using only the bitstring itself; alternatively, we add the bitstring to the entropy pool for this particular link and we wait for another round of KEx execution (possibly several rounds), to collect a sufficient amount of entropy to be able to secure given link. Given the nature of a given network, some links should be secured without any major issue, while for other links the required entropy might never be collected—all depending on the network environment, routing configuration and many other parameters. Following up on the KEx trigger, we label the links which we were able to secure only using the KEx as trusted.

The other part of our protocol is a modified secrecy amplification, also executed upon a trigger (time or event based), for a given link in the network. The trigger is deactivated for links labeled as trusted, thus saving resources required for SA execution. In addition, the SA algorithm can be modified to prefer already established trusted links in the network, improving the outcome.

The overall goal of this particular design is to save resources used for regular secrecy amplification if the network conditions are favorable for the use of key extraction. However, having the option of performing a vanilla version of secrecy amplification in the case of a network with no entropy collected by key extraction, this makes this protocol very adaptive to various environments. The expected outcome for average conditions is to secure a small portion of links using key extraction initially, and, gradually, over multiple executions of both SA and KEx triggers, producing a strong majority of trusted links in the network.

### 4.2. Entropy Crowdsourcing

Facing the issue of low entropy levels produced by the key extraction alone, in [4], we proposed two approaches to combining entropy over multiple links. These two protocols are inspired by the classical secrecy amplification protocol versions—Push and Pull. Both are described on a small subset network, composed of only three nodes—A, B, and C. We assume that all three links in between these three nodes had collected RSS measurements and successfully computed three bitstrings (noted as En), shared in between individual pairs of nodes. However, the min-entropy of any of these bitstrings does not exceed a set threshold required for a secure key For example, nodes A and B had produced a shared bitstring, but the min-entropy of such bitstring does not exceed 40 bits. Inherently, the bitstring alone cannot be used to secure the link directly.

#### 4.2.1. Push Version

**Figure 2:**
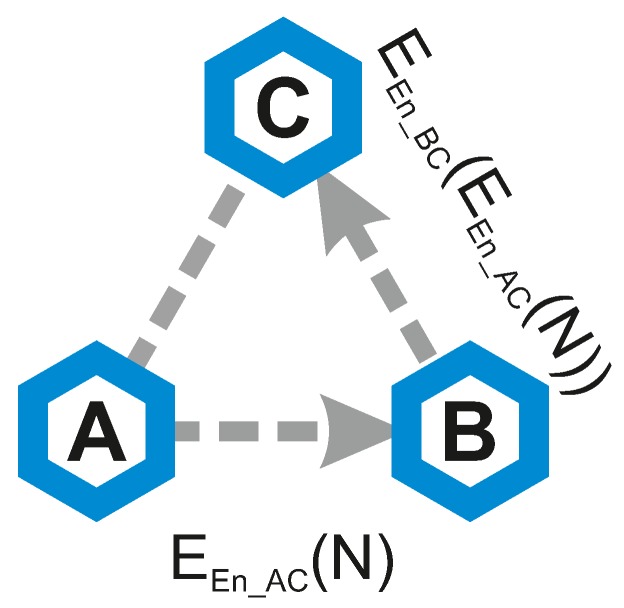
Visualisation of the Push version.

The Push version of the protocol can be described by the following steps (graphic visualization can be seen in Figure 2):

1. Node A generates a random nonce *N* to update the link key $K_{AC}$ with node C (with intermediate node B).
2. Node A encrypts the nonce *N* using the En_AC as a key and sends the resulting value $E_{En\_AC}\left(N\right)$ to node B.
3. Intermediate node B encrypts the received value using the En_BC as a key and sends the resulting value $E_{En\_BC}\left(E_{En\_AC}\left(N\right)\right)$ to node C.
4. Node C knows both En_AC and En_BC; it is able to recover the original nonce *N*.
5. Nodes A and C proceed with the key update procedure as in any other SA protocol and create a new link key $K_{AC}^{\prime }$.

#### 4.2.2. Pull Version

**Figure 3:**
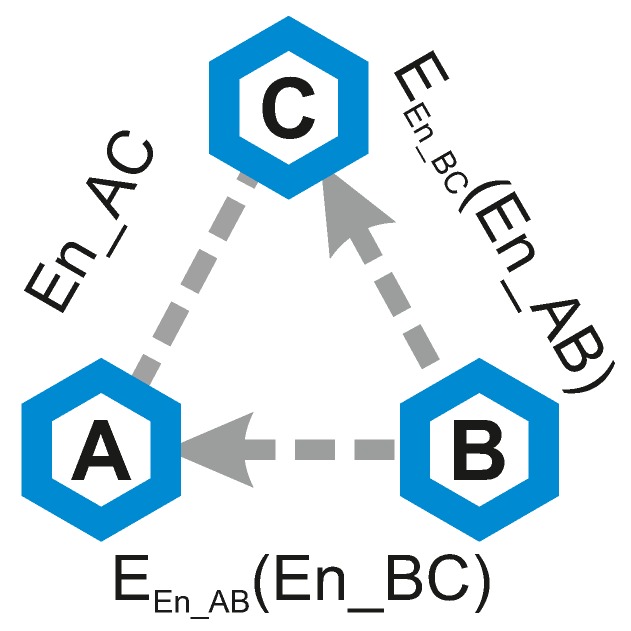
Visualisation of the Pull version.

The major difference between the Push version and the Pull version is in the initiating node. The individual steps of the Pull version are the following (graphic visualization can be seen in Figure 3):

1. Node B sends message $E_{En\_AB}\left(En\_BC\right)$ to node A. For each one of the protocol executions, we have assigned one of the three network environment settings. Having calculated the average performance of every protocol or protocol components, we then evaluated which protocol is suitable for which type of network and expected network environment:
2. Node B sends message $E_{En\_BC}\left(En\_AB\right)$ to node C.
3. Node A decrypts En_BC from the received message.
4. Node C decrypts En_AB from the received message.
5. Nodes A and C can use all three values En_AB, En_AC, and En_BC to create a key update, producing a new link key $K_{AC}^{\prime }$.

## 5. Experiment Setup

One of the main points of this article is to undertake a real testbed verification of both approaches. As such, we executed many runs of our protocols in as many different conditions as was feasible.

The KEx part of our protocols is mostly affected by the presence of people and disturbances such as other radio communication. Therefore, we selected the three following types of network environments:

- *Undisturbed network*—no people or other disturbances present in the area of network deployment. We consider the time interval during night hours and weekends, for weekdays time from 10:00 p.m. up to 6:00 a.m.—for Saturday and Sunday, we include the whole day.
- *Busy network*—peak hours, as many people and disturbances present as possible under usual conditions. We consider the time interval from 9 :00 a.m. to 5:00 p.m., as, during this time, the majority of staff members are present in the office and the majority of lectures also takes place in this time interval.
- *Normal network*—off-peak hours, some people and disturbance present, but only in limited numbers. We consider the time interval from 6:00 a.m. to 9:00 a.m., followed up by time interval from 5:00 p.m. to 10:00 p.m. Disturbances are generally present in this interval, however, only in some areas of our network.

For each one of the protocol executions, we have assigned one of the three network environment settings. Having calculated the average performance of every protocol or protocol components, we then evaluated which protocol is suitable for which type of network and expected network environment.

### 5.1. Protocol Parameters Used

For the entropy-driven secrecy amplification protocol (described in detail in Section 4.1), we defined the KEx trigger by an internal message counter so that the trigger would fire after every 100 messages. The minimal delay in between two messages was defined to be two seconds. However, due to the media access control limitations (only one node can transmit in a given time slot and location) and the actual workflow of our node, the actual time in between two messages sent on one link averaged at 4.8 s (This does not include acknowledgments. During the 4.8 s, both the actual message and acknowledgment was transmitted – more than twice the minimum time). Therefore, the hundred messages required for KEx trigger on any given link were collected after eight minutes, approximately.

The KEx trigger time interval remained almost constant for the whole experiment duration, unaffected by the network environment. This was achieved thanks to the use of an otherwise unoccupied wireless channel. Therefore, there was no other traffic generated by the presence of people during the day, which would affect the overall traffic.

As per the SA trigger, we based it on the KEx trigger count, executing the secrecy amplification either after two, four, or eight KEx triggers. We also defined two min-entropy thresholds for the SA execution – 80 bits and 128 bits of entropy. The 80-bit threshold we deem as the minimum entropy required for a symmetric key to be secure to a certain extent and the near future, while the 128 bits of entropy are considered safe without any conditions.

### 5.2. Hardware Used

**Figure 4:**
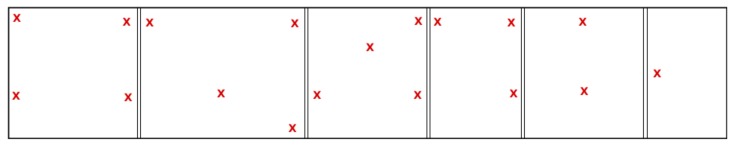
Physical placement of individual nodes in our offices; each node is represented by a red cross.

To verify our protocols beyond simulations, we opted to execute them on our laboratory testbed, containing 18 nodes. Each individual node is a single Raspberry Pi (model 3B+), running Raspbian version 9, with the kernel version 4.14.69. All nodes are connected to a single WIFI network, using the unsecured ad hoc mode, channel 48 on the frequency 5.24 GHz. Individual devices are placed on the table level across several adjacent offices, as shown in Figure 4.

## 6. Results

Results for the individual environments (environment characteristics are described in Section 5) are provided in the following subsections, while the protocol parameters are provided in Section 5.1.

### 6.1. Undisturbed Network

#### 6.1.1. Key Extraction

The absence of disturbances can be seen on the average entropy collected per individual KEx trigger, as a majority of the nodes (at least 12 out of 18 in any given experiment) did not manage to collect any entropy at all. This is due to the KEx protocol design as if the sequence of RSS measurements is constant for the whole duration of the experiment; then, no excursions are present, and zero bits are put to the output. The average min-entropy and maximum for individual nodes in this environment are presented in Table 1.

In any given experiment, up to six nodes managed to produce some entropy in the output. Out of the six nodes, at most three (two on average) produced a usable amount of entropy. The three nodes that were regularly producing a usable amount of entropy were nodes with ID numbers 6, 12, and 14. The average min-entropy estimate for any given link on these three nodes was from 10 bits up to 15 bits per KEx trigger. On a close inspection, we did not find any reason for these nodes to produce such results. Therefore, we concluded that the resulting entropy must be determined by a favorable placement of these three nodes, possibly a nearby presence of disturbances induced by other electronic devices. The remaining nodes did not manage to produce any usable output, even though they managed to measure nonconstant series of measurements. On a closer inspection of our data, we verified that indeed these nodes measured few excursions in the RSS data; however, such excursions did not occur regularly. Therefore, the resulting bit-strings had a length only up to 10 bits, and the min-entropy estimate was not measurable in most cases.

#### 6.1.2. Entropy Driven Secrecy Amplification

As can be deduced directly from the KEx results, the only SA trigger setup, which might receive enough entropy from the KEx part, such that it would not require to execute secrecy amplification, would be an SA trigger linked to eight KEx executions, with an 80-bit threshold (the longest time combined with the lower threshold). Even though on a few occasions the entropy level came quite close to the threshold (maximum min-entropy over eight KEx executions was 72.6 bits), the threshold was never exceeded. Therefore, full secrecy amplification in the vanilla version was executed on every SA trigger, as there were no trusted links present in the network.

Any other combination of SA trigger setup (two or four KEx trigger executions) and 128-bit threshold did not manage to come close to fulfilling the min-entropy requirements; therefore, as in the previous case, the vanilla version SA was always fully executed in the network.

The entropy driven secrecy amplification approach in the undisturbed network was capable of improving the security of our network, however, only due to the fallback option of vanilla secrecy amplification protocol. Regarding the consumption of resources, no energy was conserved, as in all cases we executed vanilla SA without any modifications.

#### 6.1.3. Entropy Crowdsourcing

As three nodes managed to collect repeatedly usable amounts of entropy, the entropy crowdsourcing approach can be applied to our network. The three nodes would manage to exceed half of the required entropy on five links on average (forming a small subset of our network, taking three of our nodes and few common neighbors). What links in the subset network become secured depends on the protocol selection—Push or Pull—and the sequence in which entropy threshold is exceeded on individual links. On average, we were able to secure 2.3 links in our network.

### 6.2. Busy Network

#### 6.2.1. Key Extraction

Compared to the undisturbed network environment, there were many disturbances present, affecting almost all communication channels in our network. These disturbances were caused both by the presence of people and activity of other WIFI enabled devices. Every node managed to collect usable RSS data, and the global average min-entropy estimate of produced bitstrings collected on all links was 9.5 per single KEx trigger. The overall achieved maximum was recorded on node 8, having their estimate per one KEx trigger reach 27 bits of entropy. However, the average entropy for node eight does not deviate from the global average—the maximum must have been achieved only thanks to some one-off disturbance that did not repeat.

The effects of favorable placement are still noticeable, although they are not as pronounced as in the undisturbed network. In addition, we can notice that the favorable placement changes with the environment. The effect is most pronounced on node 16—no entropy produced in the undisturbed network state, while in the busy state achieved the highest overall average min-entropy estimate of 15. The average min-entropy and maximum for individual nodes in this environment are presented in Table 1.

#### 6.2.2. Entropy Driven Secrecy Amplification

As can be seen from the overall results, the only viable SA trigger setup is linked to the eight KEx executions, which was unexpected. However, on half of the nodes, we were able to collect enough entropy to silence the SA trigger with 80-bit threshold regularly; the best performing node was even able to semi-regularly silence the SA trigger with a 128-bit threshold.

In the cases of SA trigger linked to the two or four KEx triggers (regardless of the entropy threshold), we executed the fallback option—vanilla version secrecy amplification. In the case of SA trigger connected to the eight KEx trigger executions, there were always trusted links produced by the KEx part. Therefore, we were able to silence the SA trigger on approximately half of the links in the network using the link preference during the SA execution.

#### 6.2.3. Entropy Crowdsourcing

The entropy crowdsourcing approach can be applied without any problems with a very pleasant outcome, as it directly addresses the issue of nodes being capable of collecting entropy, but only in limited amounts—this fits the four KEx trigger setup. Therefore, we limit the entropy crowdsourcing results only to a four KEx trigger setup (two KEx triggers do not come close to the usable amount of entropy, while the eight KEx triggers produce enough entropy to use KEx directly without any need for crowdsourcing). If we consider the Push protocol, which requires min-entropy over half the threshold, then nine nodes out of 18 were repeatedly able to collect more than 40 bits of entropy (assuming the threshold of 80 bits) on individual links. As the Push protocol combines entropy from two links, having half of the links to our disposal for the protocol execution, we can secure one-quarter of the links present in the network. Exact numbers highly depend on the sequence in which the entropy threshold is exceeded on individual links—which node initiates the protocol and which intermediate neighbors are used.

If we consider the Pull protocol, which combines entropy over three links, then the third of our threshold has been regularly collected on 16 nodes out of 18. Similar to the previous case, we combine entropy over three links to secure one, we manage to secure approximately 30% of the links in the network; exact numbers again depend on the sequence in which the entropy threshold is exceeded on individual links—which node initiates the protocol and which intermediate neighbors are used.

### 6.3. Normal Network

#### 6.3.1. Key Extraction

The average min-entropy estimate per single node for the normal network environment is 4.6 bits per KEx trigger, which as expected is about half of the entropy collected in the busy environment. However, the average does not apply to individual nodes. We can observe some nodes (IDs 2, 3, 6, 12, and 14) with an almost identical performance as in the busy environment, while other nodes’ performance is identical to the one in the undisturbed network. Three nodes (IDs 5, 9, and 17) did not collect any entropy at all. Therefore, we can clearly see a very pronounced effect of node placement and its direct surroundings. The average min-entropy and maximum for individual nodes in this environment are presented in Table 1.

#### 6.3.2. Entropy Driven Secrecy Amplification

The same as in all previous cases, the only viable SA trigger setup is linked to the eight KEx executions. Only three nodes were able to collect entropy required to silence the SA trigger (assuming 80-bit threshold), while most of the other nodes stayed close to the half of the required entropy.

In the cases of SA trigger linked to the two or four KEx triggers (regardless of the entropy threshold), we executed the fallback option—vanilla version secrecy amplification. In the case of SA trigger connected to the eight KEx trigger executions, there were some trusted links produced by the KEx part, although in much smaller quantity than in the busy environment. Therefore, we were able to silence only about one-eighth of SA triggers.

#### 6.3.3. Entropy Crowdsourcing

The results from KEx allow us to employ the entropy crowdsourcing approach quite well; as for the setup with four KEx triggers, as well as eight KEx triggers, we can employ both Push and Pull versions of our protocol.

The results for the setup with four KEx triggers can be considered similar to the undisturbed network with eight KEx triggers, as very few nodes produced a usable amount of entropy (in particular nodes 6, 12, and 14). The results are very dependent on the network topology, as we combine entropy over neighboring links. Thus, with this setup, we can secure only a few links without any major impact.

For the eight KEx triggers, we can see similarities to the busy network setup with four KEx triggers; however, due to the topology issues discussed in the previous paragraph (several nodes without any entropy collected), we are limited with the link and common neighbor selection, thus the overall percentage of secured links is slightly worse than in the previous case, regardless of the selected protocol. On average, we were able to secure one-fifth of links using the Push protocol and only 15% of links using the Pull protocol (even though the Pull protocol requires less entropy per link, it needs a favorable position with three links, compared to only two links for the Push version).

## 7. Conclusions

The results for individual protocols as well as the overall performance of our setup are discussed in the following subsections.

### 7.1. Key Extraction

The performance of key extraction depends on the network environment significantly, which is to be expected. The limiting factor in our case was the delay in between individual messages, as we could not accomplish the intended delay of two seconds in between two messages. Since the actual delay was more than twice as long, we have managed to collect only about half of the expected entropy from one KEx trigger on average.

The overall results correspond to our previous findings that static networks are suitable for KEx use only to a certain extent as the amount of entropy, which can be gathered, is limited (regardless of the network environment). Similar results in previous experiments motivated our research on entropy crowdsourcing [4], which proves to be still relevant, even to our current network setup.

In a busy network environment, the KEx is mostly able to work alone without any combination with other protocols; in any other setup, the KEx alone cannot re-secure the network in a reasonable time.

### 7.2. Entropy Driven Secrecy Amplification

The assumed setup of two, four, and eight KEx triggers worked only partially, as the two and four KEx triggers never produced any usable amount of entropy and consequently no trusted links. The eight KEx triggers were suitable for use with varying results from different network environments. In the busy environment, most of the nodes managed to overcome the 80-bit threshold, creating a reasonable amount of trusted links. In the normal environment, only some nodes managed to overcome the 80-bit threshold, producing way less trusted links—still, any trusted link can increase the overall performance significantly and speed up the process of securing the network completely. In the undisturbed network, we did not expect any results from the key extraction, and we were pleasantly surprised that few nodes in favorable locations produced some limited amount of entropy.

The limited amount of produced entropy also affected the 128-bit threshold, as it would require significantly more time and messages exchanged to work reliably. Therefore, for all our experiments, we had to abandon the 128-bit threshold. In addition, unexpectedly, we verified that the backup option is essential. In many cases, we were not able to collect any entropy, and the only option was to execute a vanilla version of secrecy amplification.

The results show that the entropy crowdsourcing protocol is capable of adapting to any environment, as in all of our experiments we were able to improve the security of our network.

### 7.3. Entropy Crowdsourcing

As the design for entropy crowdsourcing was motivated exactly by the conditions we were facing, the approach itself proved to be well designed. From the results of our experiments, we conclude that the Pull version of our protocol has better performance in a network with uniform entropy gathering (all links produce similar amounts of entropy). This is given by the Pull protocol design, as it requires a favorable position with three links, which can be difficult to find with uneven entropy gathering. The Push protocol, on the other hand, is capable of working in most networks, as it needs only two links. The differences in between the two were most pronounced in the normal network environment.

### 7.4. Overall Performance

Our protocols showed promising results in all possible environment setups. Using the entropy driven secrecy amplification, we were able to save 12% of the SA messages. With the entropy crowdsourcing approach, we were able to secure up to 20% of the links in the network, without any SA messages sent. To still improve the results, one would have to set longer intervals in between individual KEx triggers or allow for more than eight KEx triggers per one SA trigger. The exact setup (entropy threshold, KEx and SA triggers, and Push or Pull selection) should be defined dynamically for every network and environment, as every environment and network setup require different parameters for optimal performance.

## Figures and Tables

**Table 1:** Average and maximum entropy collected per single KEx trigger for all nodes in our network in all three environment settings (undisturbed, busy, and normal).

Node ID	1	2	3	4	5	6	7	8	9	10	11	12	13	14	15	16	17	18
*Undisturbed*																		
Avg. entropy	0	0	0	0	0	9	0	0	0	0	0	7	0	5	0	0	0	0
Max. entropy	4	9	0	4	0	14	0	0	0	0	0	13	0	11	0	0	0	0
*Busy*																		
Avg. entropy	10	6	8	11	9	11	9	10	6	7	8	11	9	12	10	15	9	11
Max. entropy	22	21	17	23	16	22	25	27	16	16	16	23	14	20	17	21	20	18
*Normal*																		
Avg. entropy	1	4	6	2	0	11	5	3	0	5	6	14	2	12	6	3	0	4
Max. entropy	13	11	12	9	0	14	7	10	0	9	11	17	6	16	10	7	0	13

## Abbreviations

The following abbreviations are used in this manuscript:

ID	Identifier
IoT	Internet of Things
KEx	Key extraction from radio channel fading
KMS	Key management scheme
RSS	Received signal strength
SA	Secrecy amplification
WIFI	Wireless local area networking
WSN	Wireless sensor network
